# Effectiveness of the school-based social and behaviour change communication interventions on insecticide-treated nets utilization among primary school children in rural Ethiopia: a controlled quasi-experimental design

**DOI:** 10.1186/s12936-020-03578-x

**Published:** 2021-01-13

**Authors:** Fira Abamecha, Morankar Sudhakar, Lakew Abebe, Yohannes Kebede, Guda Alemayehu, Zewdie Birhanu

**Affiliations:** 1 Department of Health, Behaviour, and Society, Faculty of Public Health, Institute of Health, Jimma University, P.O. Box: 378, Jimma, Ethiopia; 2 USAID/Ethiopia Office, Addis Ababa, Ethiopia

**Keywords:** Malaria, SBCC, Insecticide-treated nets, Itns, Schools, Ethiopia

## Abstract

**Background:**

School-based behaviour change communication interventions could help to achieve behavioural changes in the school and enhance the enrollment of the students and teachers as health messengers to local communities. Evidence on the impacts of the school-engaged malaria preventive interventions are limited as far as the social and behaviour change communication (SBCC) is concerned. This study examined the effectiveness of the school-based SBCC approach on insecticide-treated nets (ITNs) utilization among primary school students in malaria-endemic settings of Ethiopia.

**Methods:**

Various participatory, educational, and communication interventions were implemented from 2017 to 2019 in 75 primary schools and respective villages in Jimma to promote malaria preventive practices. A quasi-experimental design was conducted with randomly selected 798 students (i.e. 399 intervention and 399 control groups). Data were collected by trained interviewers using structured questionnaires. The SPSS version 26 software was used to analyse the data. Propensity score matching analysis was performed to control for possible confounding biases. The average effects of the intervention were estimated using multivariate general linear modelling to estimate for mean differences and odds ratio based on the nature of data.

**Results:**

The result showed that the ITNs utilization was 6.857 folds in the intervention groups compared to the counterpart; (OR = 6.857; 95% CI: (4.636, 10.1430); effect size = 39%). A mean differences (MD) of self-efficacy (MD = 15.34; 95% CI: 13.73 to 16.95), knowledge (MD = 5.83; 95% CI: 5.12 to 6.55), attitude (MD = 6.01; 95% CI: 5.26 to 6.77), perceived malaria risk (MD = 2.14; 95% CI: 1.53 to 2.76), and perceived family supports (MD = 6.39; 95% CI: 5.57 to 7.22) were observed favoring the intervention. Multivariable logistic regression modelling results showed that knowledge (β = 0.194, 95% CI: 1.09 to 1.35) and perceived family supports (β = 0.165, 95% CI: 1.11 to 1.25) and self-efficacy (β = 0.10, 95% CI: 1.22 to 2.32) predicted the ITN utilization among the school children.

**Conclusions:**

The finding of this study suggested that the school-based SBCC approach combined with peer education activities advanced the malaria-related knowledge, attitude, self-efficacy, risk perceptions, and family supports and ultimately improved the sustained use of ITNs among school-going children. Further research should be conducted to understand the mechanism of these effects given the influences of social, health services, and school systems are considered.

## Background

Malaria is one of the oldest vector-borne diseases transmitted by the bite of an infected mosquito from *Anopheles* species [1]. Malaria is an important global public health concern. A latest report shown that the global malaria cases in 2019 was 229 million and this estimate has remained unchanged over the last 4 years [2]. For instance; the disease affected 409,000 lives in 2019 compared to 411,000 in 2018 [2]. In Ethiopia; it is estimated that three-fourths of the land is below 2000 m and is malarious with two-thirds (60%) of the country’s population is at risk [3]. Peak malaria transmission occurs between September and December in most parts of Ethiopia, after the main rainy season [4]. Malaria remains a major health problem among school-aged children, affecting the critical period of learning and development [5]. The prevalence of malaria infection in school-aged children appears higher and less emphasis in malaria control has been given to this group [5–7].

Malaria elimination has received major global attention [4, 8–10] and several costly malaria control programmes such as vector control [through the use of insecticide-treated net (ITN) and insecticide residual spray (IRS)], malaria case management (screening and treatment) and improving supply chain of malaria preventive services [11] have been implemented over the decades achieving considerably good progress. However; the estimate of global malaria cases has remained unchanged over the last 4 years (i.e. from 2017 to 2020) [2]. Thus; the up-coming years are expected to demonstrate stagnation in malaria reduction; despite the planned elimination after decades (i.e. by 2030, 2050 and beyond). This signifies the need to devise effective strategy to intensifying community participatory interventions as it seems paradoxical to achieve the goal in the context of inadequate public participation [12].

One of the strategies to effectively engage community and schools in malaria preventive programmes is the use of the SBCC approach [4]. The SBCC approach is one of the emerging strategies used to advance community engagement in malaria preventive programs [9, 10]. The SBCC emphasizes an ongoing engagement and participation of the primary audience, key stakeholders and community institutions such as schools [13]. The role of schools in malaria actions that have received attention include: to support the disease surveillance, track the coverage of malaria preventive services such as ITNs use, adoption of IRS, and access to effective malaria treatments at the community-level [14]. In addition to achieving behavioural changes among school communities; the school-based health promotion practices could enhance enrollment of students and teachers as health messengers to local communities [15]. It was also suggested that school-engaged interventions have a multiplier effect in that students influence their families, neighbours, and friends towards healthy practices [16].

Although evidence is available on the effectiveness of the SBCC strategy on motivating the community towards malaria preventive behaviors in the general population [17, 18]; there was no documented evidence on how schools-engaged malaria preventive efforts influence behaviours of students as far as the SBCC is concerned. This is partly due to the recent emergence of the practice of SBCC [19, 20]. Therefore; this study examined the effectiveness of the school-based peer education approach combined with the SBCC interventions on malaria related preventive knowledge, attitude, risk perceptions, self-efficacy, and ultimately utilization of ITNs among rural primary school students in malaria-endemic settings of Ethiopia.

## Methods

### Study setting

The school-based SBCC project was implemented from 2017 to 2019 in 75 selected rural schools of Jimma zone; the State of Oromia. Jimma is located 352 km away from Addis Ababa; the capital city of Ethiopia. Based on the projected 2007 Census conducted by the CSA, the total population of Jimma zone was 2,486,155 (50.3% male and 49.7% female) and the rural population accounts for more than 89%. The zone lies within an altitude ranging between 900 and 3500 m above sea level.

The current intervention was conducted in five selected districts of Jimma Zone for intensive engagement on malaria communication. These districts were Limmu Kossa (population = 209,261), Shebe Sombo (population = 146,805), Nono Benja (population = 77,452), Chora Botor (population = 74,756), and Gera (population = 147,120). According to the Zonal health department report of 2016; an annual parasite incidence (API) rate in these districts was 16% in Chora Botor, 14.1% in Shebe Sombo, 10% in Nono Benja, 5.5% in Limmu Kossa, and 3.1% in Gera. The project was dedicated to benefiting schools and various community groups including vulnerable groups such as children less than five years, pregnant women, and school students.

### Theoretical bases to inform the school-based SBCC interventions

To guide the SBCC content and interventions process, concepts, and principles drawn from some behavioural change theories or models were combined and applied. Accordingly, the Motivation Protection Theory (MPT) and the health belief model (HBM) were used to design the proposed SBCC elements [21]. These theories explain the cognitive mediation process of behavioural change in terms of threat and coping appraisal. According to the theories, the appraisal of the health threat (i.e. health risk due to malaria in this case) and the appraisal of the coping responses result in the intention to perform adaptive responses which are called protection motivation (i.e. using malaria preventive measures, such as ITNs, IRS, prompt care-seeking, and proper use of medications).

Thus, the theories propose that the intention to protect oneself or families from certain conditions (i.e. malaria) depends upon four factors: (1) the perceived *severity* of a threatening event (e.g., a malaria attack), (2) the perceived probability of the occurrence, or *vulnerability* ( e.g. perceived vulnerability of the individual to a malaria attack), (3) the efficacy of the recommended preventive behaviour (e.g. *perceived effectiveness of recommended actions to prevent or remove the health risk, malaria in this case*) and (4) the perceived *self-efficacy* (i.e., the level of confidence in one’s ability to undertake the recommended preventive behaviour, such as regularly sleeping under ITNs, prompt care-seeking behaviour and properly using medications) [21]. To this end, formative assessment was undertaken in the target districts based on the assumption and framework of the MPT and HBM. The results obtained from this formative assessment were used to guide the malaria communication activities and to monitor behavioural change progress indicators in schools and target villages.

The second theory, the theory of diffusion of innovation (DOI), was applied to complement the individual based theories. DOI is one of the most widely used communication theories. According to the DOI, the population can be broken down into five different segments, based on their propensity and time it takes them to adopt a specific behaviour (e.g. adoption of recommended malaria prevention measures in this case). These are innovators, early adopters, early majorities, late majorities, and laggards and people in each category have different needs, perceptions and require tailored interventions [22]. In this intervention, early adaptors were considered a role model for other group members after receiving basic malaria training.

Success stories and experiences of these role models were captured and used for educational purposes to motivate other students and family remembers. People in each category of adoption need different interventions ranging from sharing simple facts to implementing groups’ norms [22]. Thus, the due emphasis was given on promoting social and groups’ norms rather than just the health benefits of interventions and emphasizes the risks of being left behind for those who are late and laggards to adopt the behaviours. Thus, the group members reinforce each other, and households who do not practice the recommended behaviour begin to model a new behaviour and change themselves as a result of pressure from the group members and social networks. Household status and student behaviours were monitored and evaluated, and tailored education was provided accordingly.

### Descriptions of the intervention

The current school engaged peer education combined with SBCC intervention was designed to facilitate behaviour changes on malaria prevention and control targeting various levels of personal, organizational, and community factors. Ultimately, it was intended to promote the five key malaria prevention and control practices both at schools and community levels. These were the use of insecticide nets (ITNs), appropriate & timely seeking care for malaria, appropriate use of quality anti-malarial drugs, acceptance of insecticide residual spray (IRS), and draining of potential breeding sources in the villages.

**Fig. 1:**
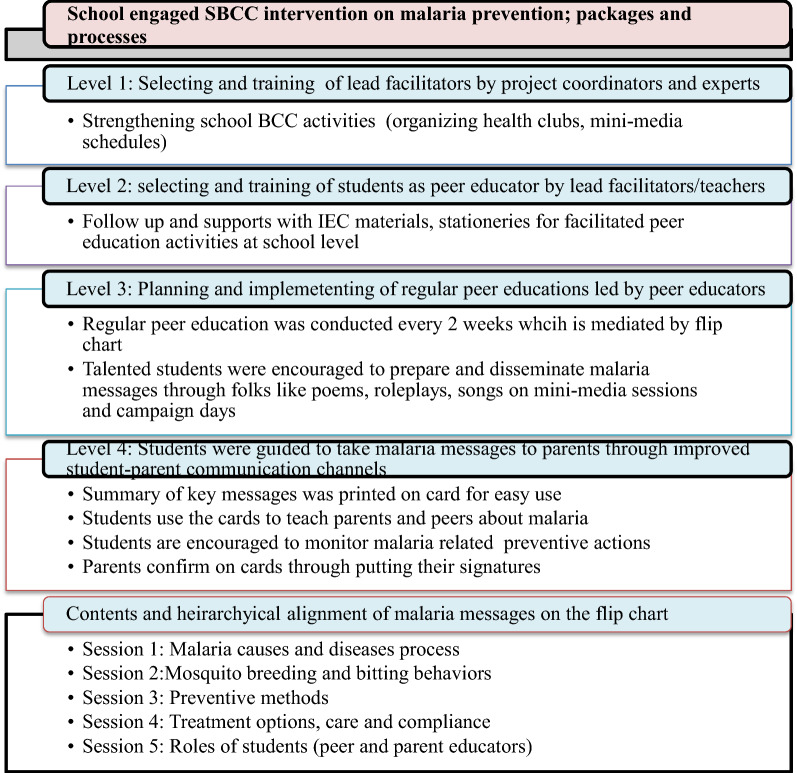
Summary of school engaged SBCC intervention packages and process

The interventions encompassed various capacity building and educational sessions that were implemented from 2017 to 2019 engaging 75 schools and respective villages. The programme was first initiated through participatory consultations of stakeholders or representatives of the community including key peoples from health offices, education offices, health extension workers (HEWs), and village leaders and schools. The formal supervisory committee was organized before the actual joint situation analysis that identified malaria situations, the interventions' needs, and strategies. Based on the need assessment results, joint planning (i.e. identifications of roles, developing goals/objectives, devising monitoring, and evaluation mechanisms). Finally, the plan was implemented over two and a half years through active engagement of the community, health institutions, and primary schools. A summary of the intervention process is presented in Fig. 1. Furthermore, details about the intervention are provided in another publication [23].

### Study design

The study employed a quasi-experimental evaluation design to collect post-intervention data from selected primary school students (i.e. grade 6th through grade 8th). A post-intervention quasi-experimental design was most widely and effectively used in impact evaluation of large scale interventions [24]. Students in intervention schools were considered exposed (intervention group) and those selected from non-intervention schools are comparison/control group. Controls were selected from adjacent schools to the project area of the same cluster.

### Study populations and sample size

All grades 6th through 8th students in randomly selected schools from both the intervention and controls were considered to study population and included in the study. The sample size was calculated using two population proportion [25] given by $n=\frac{\left(P\right)\left(1-P\right)\left(Z\alpha +Z\beta \right)2 }{E2} [\frac{r+1}{r}]$ where $P=\frac{P1+P2}{2}$, *E* = *P*_*1*_*-P*_*2*_ and *r* = the ratio of the two proportions. It was assumed to detect the effect or odds ratio two (OR = 2 or greater) for 90% power (Z_β_ = 0.96) and 5% level of significance (Z_α/2_ = 1.96) with an equal number of intervention and comparison groups. The P_2_ = population prevalence of ITNs use among school children was 46% which was taken from the previous study [26]. This yields a sample size of 380 (190 each intervention and control groups) was calculated. Considering a factor of 2 for sampling variation or design effect and 5% for non-response rate, the final sample of 798 (399 each intervention and control groups) was drawn.

### Sampling techniques

**Fig. 2:**
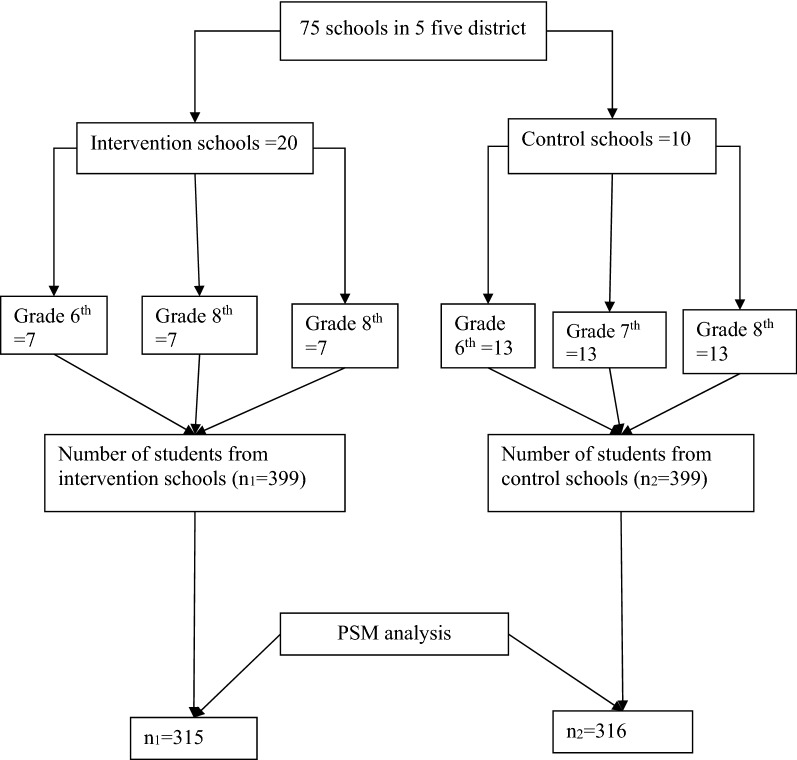
Sampling techniques and PSM approach

Seventy-five schools in five districts were addressed by the intervention. A total of 4 schools per each district (i.e. 4*5 = 20 schools) were randomly selected from the intervention village. Two corresponding schools from non-intervention village (i.e. 2*5 = 10 schools), but in the same district were randomly selected. Stratification was further done to distribute a 399 sample to each school and grade levels through grades 6th to 8th assuming an equal number of students in all schools. This was done by dividing 399/20 for intervention and 399/10 for control, which gives 20 and 39 students per school respectively. Down stratifying to grades level, 6th through 8th = 3, (i.e. 20/3 = 7 and 39/3 = 13) for intervention and non-intervention schools respectively. Finally, 399 students each from 20 interventions and 10 non-intervention schools were interviewed (Fig. 2).

### Data collection tools and methods

Malaria related data were collected using a questionnaire adapted from relevant literature such as malaria indicator survey and health, and demographic survey [27, 28]. The questionnaire form covered socio-demographic factors, peer education experiences, behavioural outcomes such as ITN use, and psychographic outcomes such as knowledge, risk perceptions, self-efficacy, and attitude related to malaria and its preventive measures. The questionnaires were then translated into the local language, *Afan Oromo,* before data collection. Data was collected on an interviewer-administered basis. Qualified data collectors recruited ad three days of training were given to data collectors and supervisors about the purpose of the study, instruments, and data collection procedures. The data collection process was closely supervised by the research teams. Eligible students were called on by schools’ administrators to appropriate places for the interview which was conducted face-to-face.

### Variables and measurements

The current study measured two outcomes of interest and these were measured at the end of the intervention. The primary outcome of interest was the ITNs utilization. The secondary outcomes of interest were the psychographic outcomes that include multidimensional knowledge, attitude, family supports, self-efficacy, and perceived malaria risk and severity. The psychographic outcomes or variables are conceptualized in this study as mental processes such as attitude, perceptual process, and beliefs about an individual’s behaviours or practices in the context of malaria preventive behaviours.

#### Knowledge

Multidimensional knowledge (MDK) questionnaire was used to measure comprehensive malaria knowledge related to the cause of malaria, signs/symptoms, vulnerable groups, preventive measures, and mosquito vectors biting behaviours. Questions encompassing 31 items were used [29]. For each item, the correct answer was assigned (1 = Yes) and an incorrect answer as (0 = No). Scores of correct responses were computed for subsequent analysis.

#### Attitude

This is evaluative beliefs or acceptance and benefits towards the ITN, indoor residual spray, vulnerable groups, and malaria situation in the study area. The items were scored on a five-point Likert scale ranging from *strongly disagree (1)* to *strongly agree (5).* The reliability statistics or internal consistency of the items were equals, α = 0.72. The overall attitude score was computed by summing up the items after performing the reverse scoring for negatively worded sentences. A higher composite score indicated a more favorable attitude.

#### Perceived malaria risk

This was defined as individuals’ perception of vulnerability to malaria in the context of their daily experiences about the presence or absence of malaria, individuals who suffered from malaria in the villages. It was measured using seven items on a five-point Likert scale, (α = 0.76). A higher composite score was interpreted as a higher perception of risk.

#### Perceived severity of malaria

This represented the perceptions of bad consequences of the malaria infection in causing pain, death, interruption with daily activities such as schooling, and its impact on their academic performances. Four items were used to tap this construct by using a five-point Likert scale, (α = 0.84). Reverse scoring was done for both perceived risk and severity before conducting further analysis**.**

#### Self-efficacy

Self-efficacy is defined as an individual’s confidence or beliefs about the ability to prevent themselves from malaria infections under given circumstances by using ITNs or any other preventive measures. It addresses aspects of malaria prevention such as using ITN, application of IRS despite its perceived discomforts, ITN handling, early care for fevers and drug adherence, etc. Four items were used to measure it. Scoring and computing were all were done the same way. The items showed an acceptable level of internal consistency with α = 0.89.

#### Perceived family support

This was conceptualized as perceptions about how much their family (parents and siblings) are motivated to use ITNs or IRS as preventive measures, advice and encourage the individuals on ITN access and use, and help them handle the ITNs for effective and sustained use. Ten items were used to measure this dimension using five-point Likert scales. The measure of internal consistency or reliability statistics was acceptable level, α = 0.84. A higher score was interpreted as strong perceived family supports.

#### Utilization of ITNs

The access to, and use of ITNs was assessed by three items that include the presence of ITNs in the home, number of ITNs, and ITN utilization every night. It was coded as *Yes* = *1* if the student used the net every night and otherwise *No* = *0.* Access to ITNs (ratio of ITNs) was defined as the presence of at least one ITN per two individuals in the household [8].

#### Social desirability bias (SDB)

Is defined as a tendency to portray oneself in a socially desirable manner [30]. In this study, it is believed that the SDB might exist especially among respondents in intervention schools as they were aware of the aim of the intervention. They could respond to the interview in a socially desirable manner; thus hiding reality. Consequently, it was interesting to measure the SDB to analytically adjust for its confounding effects on the outcomes. This was measured using the SDB for children scale that consists of 20 items constructed such that Yes = 1 if the condition exists and No = 0, otherwise [30]. The continuous score was computed from all 1′s (correct) responses. A high score was interpreted as the presence of a high SDB. This score was adjusted in every subsequent analysis and considered during the interpretation of the result.

### Statistical data analysis

Data were analysed using statistical package for social sciences (SPSS) version 26 software for analysis. Means, standard deviations, frequencies, and proportions were calculated as descriptive analysis. The propensity score matching (PSM) technique was performed as a matching analysis to reduce the selection bias due to a lack of randomization in this study. The PSM was recommended in evaluation studies where observational data are used to adjust for the possible differences between intervention and control groups at baseline [31]. The method was found effective in reducing biases in observational and quasi-experimental studies [32]. A study conducted to evaluate the effectiveness of community-based SBCC interventions on the use of ITNs demonstrated that PSM approached was productive [33].

The propensity scores were predicted or computed based on the selected covariates or characteristics that may lead to the possible differences at baseline. Assuming that there are no unobserved differences between the groups (i.e. satisfying assumptions of ignobility), all socio-demographics and socio-economic predictors were included in the matching procedure that are known to be associated with both intervention assignment and the outcome [32]. Accordingly, ten covariates that include age, gender, grade point average (GPA), grade level, ethnicity, religion, schools, specific roles of the student in the class (e.g. being leader, deputy leader or member), and participation in school wide-clubs (e.g. sport club, health club, technology clubs) and specific roles in the clubs (e.g. being leader, deputy leader or member) were selected to compute propensity scores.

Multivariable logistic regression modelling was used to estimate the propensity scores. The test of normality of the predicted scores was done to evaluate the balance of the matching result for the goodness of model fit. The matched sample was used in the subsequent analysis. Participants were matched using the one-to-one nearest neighbour algorithm by imposing tolerance level or calipers of width equal to 0.20 of the standard deviation of the estimated propensity scores [34]. Individuals scores not falling within this specified distance were excluded from the sample [31].

The average effect of the intervention on psychographic outcomes (including knowledge, attitude) was estimated using multivariate general linear modelling. The adjusted mean differences and effect size was calculated to examine the effects of the interventions on these psychographic outcomes. An odds ratio was calculated to estimate an average effect of the intervention for binary outcomes (e.g. ITNs utilization). The analysis was adjusted for the confounding effects of the SDB and predicted propensity scores. The predicted propensity score was included in the subsequent analysis to adjust for the covariates it has been represented in matching analysis. Covariates that were included in the estimation of propensity score were excluded from this analysis. A p-value of less than 5% was considered to carefully signify the presence of association or difference.

## Results

### Socio-demographic characteristics and PSM analysis

A total of 709 individuals participated, with a response rate of 89% (50.2% *versus* 49.8% for control and intervention groups, respectively). The subsequent analysis was done with 631 matched samples resulting from PSM analysis. For matched sample, the majority of the participants were the age fewer than 14 years and grade seven accounts large proportion for both intervention and control groups (34.3% *vs* 34.5%). A large number of participants from control and intervention groups have a GPA of less than 73 and greater than 89, respectively. The favorite subject for the majority of control was *Afan Oromo* (the local language) as math was for the intervention group. The minority of the participants: 48 (15.2%) and 64 (20.3%) from control and intervention have leadership roles, respectively (Table 1).

**Table 1:** Sociodemographic characteristics of primary school students before and after matching analysis in Jimma, Ethiopia, 2019 (N = 631)

Characteristics	Prior matching (n = 799)			After matching (n = 631)		
	Control, n (%)	Intervention, n (%)	p-value	Control, n (%)	Intervention, n (%)	p-value
Age						
< 14 years	204 (28.8)	182 (25.7)	0.125	179 (56.6)	161 (51.1)	0.1000
> 15 years	152 (21.4)	171 (24.1)		137 (43.4)	154 (48.9)	
Gender						
Male	181 (25.5)	189 (26.7)	0.472	161 (50.9)	165 (52.4)	0.9820
Female	175 (24.7)	164 (23.1)		155 (49.1)	150 (47.6)	
Religion						
Muslim	241 (34.0)	223 (31.5)	0.041	231 (73.1)	203 (64.4)	0.9421
Orthodox	77 (10.9)	70 (9.9)		60 (19)	63 (20)	
Protestant	38 (5.4)	60 (8.5)		25 (7.9)	49 (15.6)	
Ethnicity						
Oromo	290 (40.9)	319 (45.0)	0.001	251 (79.4)	283 (89.8)	0.1000
Amhara	48 (6.8)	20 (2.8)		47 (14.9)	19 (6)	
Others (Kefa, Guraghe)	18 (2.5)	14 (2.0)		18 (5.7)	13 (4.1)	
Grade level						
Grade 6th	115 (16.2)	108 (15.2)	0.831	106 (33.5)	95 (30.2)	0.9564
Grade 7th	122 (17.2)	120 (16.9)		109 (34.5)	108 (34.3)	
Grade 8th	119 (16.8)	125 (17.6)		101 (32)	112 (35.5)	
GPA per quartile						
< 73	110 (15.5)	73 (10.3)	0.001	104 (32.9)	64 (20.3)	0.1000
73–81	92 (13.0)	80 (11.3)		84 (26.6)	70 (22.2)	
81–89	87 (12.3)	96 (13.5)		75 (23.7)	83 (26.3)	
> 89	67 (9.4)	104 (14.7)		53 (16.8)	98 (31.1)	
Favorite subject						
Biology	42 (5.9)	39 (5.5)	0.006	38 (12)	34 (10.8)	0.1000
Chemistry	41 (5.8)	44 (6.2)		36 (11.4)	40 (12.7)	
Mathematics	64 (9.0)	86 (12.1)		53 (16.8)	78 (24.8)	
Geography	44 (6.2)	46 (6.5)		42 (13.3)	39 (12.4)	
English language	28 (4.0)	39 (5.5)		25 (7.9)	33 (10.5)	
*Afan Oromo* (local language)	104 (14.7)	59 (8.3)		92 (29.1)	53 (16.8)	
Civic and ethical education	33 (4.7)	39 (5.5)		30 (9.5)	38 (12.1)	
Roles in the class						
Leader	54 (7.6)	72 (10.2)	0.043	48 (15.2)	64 (20.3)	0.9753
Vice leader	45 (6.4)	238 (5.9)		43 (13.6)	34 (10.8)	
Members	356 (36.3)	352 (33.6)		225 (71.2)	2 17 (68.9)	
Participation in school wide clubs						
Sport	75 (14.0)	78 (14.5)	0.010	56 (12)	75 (16.1)	0.9145
Health	9 (1.7)	83 (15.5)	0.000	9 (1.9)	65 (14.1)	0.1000
Mini-media	7 (1.3)	21 (3.9)	0.094	4 (0.9)	20 (4.3)	0.8723
Technology	2 (0.4)	8 (1.5)	0.191	1 (0.2)	6 (1.3)	0.9841
Environmental health	2 (0.4)	15 (2.8)	0.015	2 (0.4)	13 (2.8)	0.1000
Language and culture	6 (1.1)	22 (4.1)	0.039	4 (0.9)	20 (4.3)	0.9284
Gender equality	74 (13.4)	82 (15.3)	0.0312	58 (12.5)	78 (16.8)	0.9870
Roles in the clubs						
Leader	6 (1.2)	32 (6.6)	0.0015	5 (1.2)	28 (6.8)	0.1000
Vice leader	4 (0.8)	12 (2.5)		3 (0.7)	11 (2.7)	
Members	157 (32.4)	255 (52.6)		121 (29.2)	230 (55.6)	

### Peer learning and education activities in schools

The majority 65 (87.8%) of students in the intervention schools have a membership to health-related or malaria clubs. The highest number of participants from intervention schools had leadership roles: 133 (66.5%). Peer education activities on malaria issues were one of the activities components of school clubs. A considerably large number of students from intervention: 282 (54.4%) and control, 236 (45.6%) have reported they conducted peer education schedule every week. The majority, 108 (55.7%) and 86 (44.3%) for intervention and control schools, respectively, reported the peer education activities were self-initiated (by students). In the same way, the majority (238 (61.7%) of participants from the intervention group reported a high degree of participation among members during the peer education sessions. A low level of participation was reported by the majority of control participants, 143 (23%).

Regarding exposure to malaria information, a large number of participants from both intervention; 306 (52.8%) and control; 274 (47.2%) were exposed to malaria-related information from different sources. Specific to sources of information, the highest [161 (93.6%)] and lowest [11 (6.4%)] exposures rate to information from peer educators' were reported among intervention and control groups, respectively. The same thing is true for teachers, community, and mass media with 135 (91.8%) and 80 (22.3%), 31 (68.9%) and 14 (31.1%), and 68 (87.8%) and 21 (23.6%) in control and intervention groups, respectively (Table 2).

**Table 2:** Main sources of malaria information and peer learning and education activities among primary school students in Jimma, Ethiopia, 2019 (N = 631)

Engagement in PE activities	Control, n (%)	Intervention, n (%)	p-value
Membership to malaria/health club			
Yes	9 (12.2)	65 (87.8)	0.001
No	166 (42.8)	222 (57.2)	
Roles in any clubs in the schools			
Leader	57 (33.5)	133 (66.5)	0.001
Secretary	56 (51.4)	53 (42.3)	
Member	203 (57.7)	149 (42.3)	
Frequency of peer education sessions	N1 = 280	N2 = 300	
Within week	236 (45.6)	282 (54.4)	0.001
> 2 weeks	80 (700)	33 (29.2)	
Initiation of peer discussion			
Student self-initiated	86 (44.3)	108 (55.7)	0.039
Teachers initiated	19 (49.7)	193 (50.3)	0.509
School principal imitated	34 (54.6)	28 (46.4)	0.293
Participation in peer discussion			
Low	168 (27)	73 (11.3)	
Active	143 (23)	238 (61.3)	0.001
Ever exposed to malaria information			
Yes	274 (47.2)	306 (52.8)	0.001
No	39 (95.1)	2 (4.9)	
Sources of malaria information	n_1_ = 274	N_2_ = 306	
Peer educators	11 (6.4)	161 (93.6)	0.001
Team member	12 (8.2)	135 (91.8)	0.001
Teachers	80 (22.3)	278 (77.7)	0.001
Community^a^	14 (31.1)	31 (68.9)	0.006
Mass media^b^	21 (23.6)	68 (87.8)	0.001
Message contents (major)			
About cleaning around house	73 (38)	119 (62)	0.006
About sleeping under ITN	77 (26.7)	211 (73.3)	0.008
About care-seeking for fever	20 (14.9)	114 (85.1)	0.001
About adherence to AMD’s	27 (22)	96 (78)	0.006
About handling ITN (washing, repair)	9 (13)	60 (87)	0.001
About vulnerable groups	38 (21.1)	142 (78.9)	0.001
Safety precaution for IRS	7 (10)	59 (90)	0.001
I don’t know	42 (31.5)	97 (68.5)	0.001

^a^Community = community meeting, campaign, religious leaders, etc

^b^*Mass media* TV/radio, posters, billboards, *PE* peer education,b p-value is significant at 0.05

### Effects on malaria-related psychographic outcomes

The results showed a mean differences (MD) of self-efficacy (MD = 15.34; 95% CI 13.73 to 16.95), comprehensive knowledge (MD = 5.83; 95% CI 5.12 to 6.55), attitude (MD = 6.01; 95% CI 5.26 to 6.77), perceived malaria risk (MD = 2.14; 95% CI 1.53 to 2.76), and perceived family support (MD = 6.39; 95% CI 5.57 to 7.22) favoring the intervention group. But the finding showed a lower mean score for the perceived severity of malaria (MD = − 3.58; 95% CI − 4.16 to − 3.00) among intervention groups. Further, comparatively, the highest and lowest impact of the intervention (effect sizes) was reflected on self-efficacy (ES = 31%), and perceived malaria (ES = 7%)(Table 3).

**Table 3:** Multivariate general linear modelling parameters for the effects of the school-based SBCC intervention on malaria-related psychographics and behavioral outcomes among primary school students in Jimma, 2019 (N = 631)

Psychographic outcomes	Mean (SD)			
	Intervention	Control	MD (95% CI)	Effect sizes
MDK score	15.03 (4.55)	9.00 (4.03)	5.83 (5.12 to 6.55)	0.30
Attitude	26.02 (3.92)	20.93 (5.39)	6.01 (5.26 to 6.77)	0.28
Self-efficacy	55.76 (7.71)	42.26 (11.82)	15.34 (13.73 to 16.95)	0.31
Perceived risk	19.26 (3.72)	17.70 (3.85)	2.14 (1.53 to 2.76)	0.07
Perceived severity	13.48 (3.99)	16.45 (3.11)	− 3.58 (− 4.16 to − 3.00)	0.19
Family support	25.04 (4.01)	19.38 (5.91)	6.39 (5.57 to 7.22)	0.26

Behavioral outcomes	Intervention, n_1_ (%)	Control, n_2_ (%)	Effects; OR	95% CI
Presence of at least one ITNs in the household	n_1_ = 313	N_2_ = 316		
Yes	307 (98.1)	299 (94.6)	2.909	(1.132, 7.479)
No	6 (1.9)	17 (5.4)	–	–
Ratio of ITN per 2 persons	N_1_ = 308	N_2_ = 304		
≤ 1 ITNs	165 (53.6)	151 (49.7)	0.855	(0.623, 1.175)
> 1 ITNs	143 (46.4)	153 (50.3)	–	–
ITN of utilization	N_1_ = 308	N_0_ = 300		
Yes	264 (85.7)	140 (46.7)	6.857	(4.636, 10.1430
No	44 (14.3)	160 (53.3)	–	–

*SBCC* social and behaviour change communication, *MDK* multidimensional knowledge, *SD* standard deviation, *MD* mean differences

**Fig. 3:**
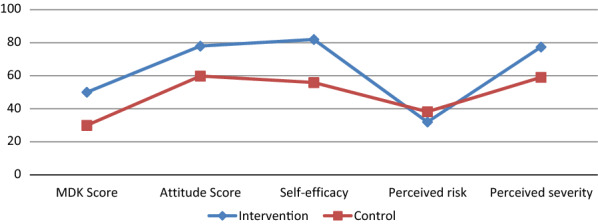
Malaria related pschographics outcomes profile for intervention and control group among primary school students in Jimma, 2019

The comparison of the psychographics outcomes profile between the intervention and control groups was graphically presented using (Fig. 3). Except for perceived severity, the graph showed that the curve for the intervention group is consistently aligned above and over that of the control group indicating the intervention group has higher scores of the remaining psychographic outcomes. On the contrary, perceived severity scores were seen higher for control in this study indicating the reduction in this construct due to exposure to the intervention. Knowledge is generally found lower for both groups compared to other variables. As indicated there was no difference in SDB scores between the intervention and control groups.

### Effect of the intervention on ITN Utilizations

The majority of both the intervention and control groups (98.1% and 94.6%) reported the presence of at least one ITN in the household. Respondents who reported having at least one ITN in the household is nearly three times higher in the intervention group (OR = 2.909; 95% CI 1.132, 7.479). There was not a considerable difference in ownership of more than one ITN per two family members between the intervention and control groups (OR = 0.855; 95% CI 0.623, 1.175). The ITNs utilization was 6.857 folds in the intervention groups compared to the counterpart (OR = 6.857; 95% CI 4.636, 10.1430); effect size, ES = 39%). This implies the ITNs use was 39% higher in the intervention group which was attributed to the intervention (Table 3).

### Independent predictors of the ITNs utilization

Multivariable logistic regression modelling (adjusted for predicted propensity score and intervention status) were conducted to examine how psychographic dimensions interacted with each other to affect the primary outcome of the intervention (i.e. ITN utilization among primary school students). Accordingly, knowledge (MDK), self-efficacy, perceived family support, peer educators as main sources of information, and the ratio of ITN in the household were predicted the ITN utilization.

This implies that students who reported having one ITN per two family members were nearly two times more likely to use ITNs (AOR = 1.76, 95% CI 1.01 to 3.07). Higher scores of knowledge (β = 0.194, 95% CI 1.09 to 1.35) and perceived family support (β = 0.165, 95% CI 1.11 to 1.25), and self-efficacy (β = 0.10, 95% CI 1.22 to 2.32) were associated with improved use of ITNs among students. However, important factors in the health behaviour change process such as attitude (β = − 0.03, 95% CI 0.90 to 1.05), perceived disease risk (β = − 0.004, 95% CI 0.96 to 1.05), and severity (β = − 0.07, 95% CI 0.99 to 1.16) were not predicted ITNs use among students (at p-value < 0.05). Those who had adequate access to the nets and thought they had greater family support were more likely to use it and vice versa (Table 4).

**Table 4:** Multivariable logistic regression modelling for the independent predictors of ITNs use among primary school students in Jimma, 2019 (combined sample, N = 631)

Predictors	Beta (β)	AOR	95% CI	p-value
Intervention status				
Control	Ref	Ref	Ref	Ref
Intervention	0.95	2.58	[1.53, 4.34]	0.001
Predicted propensity score	− 0.001	1.70	[0.29, 9.96]	0.304
Sources of malaria information				
Peer educators	0.96	2.60	[1.30, 5.22]	0.002
School teachers	− 0.27	0.76	[0.36, 1.61]	0.477
Mass media^a^	− 0.50	0.61	[0.29, 1.26]	0.180
Community^b^	1.25	3.49	[0.89, 13.74]	0.074
Ratio ITNs per 2 persons in the home				
≤ 1 ITNs	Ref	Ref	Ref	Ref
> 1 ITNs	0.57	1.76	[1.01, 3.07]	0.045
Frequency of peer education sessions				
≤ 2 weeks	Ref	Ref	Ref	Ref
≥ 2 weeks	− 0.16	0.86	[0.42, 1.73]	0.665
Knowledge	0.194	1.22	[1.09, 1.35]	0.000
Attitude towards preventive measures	− 0.03	0.97	[0.90, 1.05]	0.478
Self-efficacy preventive measures	0.100	1.10	[1.08, 2.12]	0.003
Perceived family support	0.165	1.18	[1.11, 1.25]	0.000
Perceived malaria risk	0.004	1.004	[0.96, 1.05]	0.864
Perceived malaria severity	0.071	1.074	[0.99, 1.16]	0.079

^a^Mass media include (TV, radio, billboard, posters)

^b^Community includes (religion, community gatherings, meetings), Significance was considered at p < 5%

## Discussion

This study was intended to evaluate the effectiveness of the SBCC approach to the utilization of ITNs as malaria preventive practices in primary schools in rural Ethiopia. This study was supposed to be the first of its kind in Ethiopia that had evaluated the integrated school-engaged and SBCC interventions on malaria preventive practices among rural primary school students. The study demonstrated improved malaria-related psychographic outcomes (i.e. knowledge, attitude, risk perceptions, self-efficacy) and the utilization of ITNs among target students, indicating the effectiveness of the interventions.

Knowledge is considered a basic factor in the process of health behaviour change efforts. The knowledge score which was measured as knowledge was considerably higher among the intervention groups in our study. A study conducted in India to evaluate the effects of multiple behaviour change approaches (i.e. health education plus distribution of learning materials versus only learning materials) showed significant improvement in knowledge by 21% among students in the intervention schools [35]. In Mali, school-based malaria education and distribution of ITNs indicated increased malaria-related knowledge and net use in intervention schools [36]. Furthermore, evaluation studies of school-based participatory health education in Mali and Ghana showed significant improvement in knowledge about ITN, cause of malaria, and preventive measures among school children [16, 37]. This proves that the importance of health education to reduce misconceptions about malaria thereby improving health literacy [16].

Having led to a improvement in perceptions of disease risks, attitudes, and self-efficacy towards malaria preventive actions, the current interventions justified the notions of cognitive-behavioural theories [21]. Thus, it implicated efforts targeted at enhancing the threat perceptions and attitudes with balanced coping skills would be fruitful in such behaviour change intervention efforts. However, the intervention resulted in a reduced level of perceived severity of malaria in intervention groups compared to the intervention one. This implies that the students in the SBCC target schools tend to hold lower beliefs of malaria risks compared to their counterparts. This finding contradicted the principles of many behavioural theories which claimed individuals who have a higher perception of severity or consequences of the disease are more likely to follow a certain course of preventive actions [21, 38, 39].

The possible reasons for this paradoxical result may be due to the improvement in self-efficacy and social supports on ITN utilization among the intervention groups. This finding is appears consistent with the result of a community-based study conducted to evaluate the same programme (i.e. the school-based SBCC approach), which indicated the reduction in the community’s attitude (− 3.5%) and perception of risk (− 3.3%) regarding malaria [23]. The aforementioned study was conducted among parents while the participants of the current study are students. However, this interesting relationship must be further investigated.

Family participation in school health programmes was considered one of the principal components of the Health Promoting Schools policy [40]. The theory of communication indicated that targeting social norms and support in behaviour change intervention reinforces behaviour to sustain [39]. Engaging parents was one of the key components of the current intervention aimed to enhance family supports for improved adoption and sustained malaria prevention in the community and schools. Consequently, the result showed higher mean scores in perceived family supports among the intervention group. The improved communication between parent and students on malaria prevention in this study might have impacted the perception of family influence in the intervention groups [35]. Furthermore, this effect might also be attributed indirectly to the fact that improved parental knowledge and attitude from malaria education by HEWs at-home visits.

Further, the primary outcome of interest in the current study was changes in the utilization of the ITNs in the intervention targets. In principle, the observed changes in the levels of psychographic and social supports outcomes should be translated into actions or behaviours [21]. The result demonstrated that substantial differences in the percentage point of 39% in ITNs utilization between the two groups. Similar findings were reported from previous evaluation studies of school-based interventions [35, 41]. Different levels of impact on net use were reported from previous studies conducted in India (22.2%) and Mali (41%) [35, 36]. However; the contradictory finding was reported from other studies conducted in Ghana with no changes in malaria preventive practices such as using ITN in the intervention schools [16].

The possible difference might be attributed because of integrated peer education networks and community-led SBCC approaches in the current programme. Parents were actively participating in the programme through what is locally called “*geengoo qulqullina barnootaa*” which means the circle for quality of education. The circles for the quality of education are made of members representing the community, teachers, students, and accountable to school directors have the ultimate task of improving the quality of education. More importantly, the community-based evaluation study conducted enrolling parents indicated improved acceptance of the intervention that employed students as messengers of malaria information [23].

The multivariable logistic regression modelling identified knowledge, self-efficacy, family supports, peer educators as sources of information, and the ratio of ITNs in the household as independent predictors of ITN utilization among school students. Access to more than one ITN per two persons in the household independently predicted the use of ITN in this study. A similar finding was reported from a previous school-based study in that ownership was found as the main predictor of ITN use [29]. Ensuring access to the ITNs cannot guarantee its use since several underlying factors such as resources, money and family support contexts affect the behaviours of school children. For instance, a study indicated that the net use behaviours were low among school-aged children than the rest of the population [41]. These factors are mainly classified as behaviour-driven and access-driven non-use [42]. With behaviour-driven factors, the current study identified knowledge, family supports, and self-efficacy as factors influencing the use of ITNs among school children which is consistent with that of previous studies [29, 43].

In the previous study, the total knowledge index was associated with both ITN ownership and its use. However, the study reported varying levels of influence for different domains of knowledge [29]. In short, the finding implies intervention targeting comprehensive knowledge would produce effective behaviour change outcomes regarding malaria prevention in schools. On the other hand, self-efficacy, the individual perception of confidence to undertake specific health behaviour (ITN use in this case), was associated with ITN use in this study. Notably, self-efficacy has a strong theoretical basis in social cognitive theory [39] and has been identified as the strongest predictor of health behaviours [38].

Though evidence is scarce in this regard; the implication of the current study could be explained by the notion of cognitive-behavioural theories. Given that adequate knowledge, a higher tendency of perceived coping responses to certain health risks (i.e. malaria infection in this case) can lead to behaviour change [21]. However, in this study, perceived ability should be interpreted with precaution as access to enabling factors, such as ITNs, might hamper the translation of individuals’ confidence and readiness into action.

Perceptions about family support independently predicted the use of ITN among school children in this study. Effective behaviour change interventions require enhancement of social support and this is the ultimate goal of SBCC strategy which influences community norms, social factors, and reinforcements to bring about sustained behaviour change [39]. Families are considered the immediate persons to influence their children and their support could reinforce behaviours. The observed family influence in this study might be due to the improved parent-student communication in response to malaria messages and education offered to parents by students. Further, the indirect influence might be attributed to malaria education by HEWs during home visits [23]. The finding implies emphasizing knowledge, self-efficacy while addressing social support would be fruitful to improve ITN use in primary schools.

The analysis has evaluated the effects of various sources of malaria information such as peer educators, teachers, mass media, and community (interpersonal) on ITN use among students. Among these various sources reported in this study; peer educators were found positively associated with the use of ITN in this study. This might be because of greater attention and opportunity for open discussions that result from perceived age similarity among the students and the length of time they spent together [44]. The school-level small social network among students could help the promotion of normative changes through behavioural modelling and facilitated sharing of success stories, experiences, and simple facts during educational sessions which were accomplished in the current intervention. Evidence suggested the importance of this approach to promoting social and groups’ norms rather than just the health benefits of interventions and emphasizes the risks of being left behind for those who are late to adopt the behaviours [22].

The relationship between ITN use and important factors such as attitude and perceptions of disease threat was not importantly high in this study and this contradicts the principles of many behavioural change theories [38]. Specific to attitude, the possible reasons behind this fact can be explained in many ways. First, the observed effects of self-efficacy and family support on ITN use in this study might have underestimated or masked the roles of attitudes. Evidence showed that attitude-behaviour consistency is influenced by a condition called *situational constraints* related to the presence of external factors. *Situational constraints* are circumstances that affect (through moderation) the relationship between attitude and behaviours [45]. Accordingly, strong associations between behaviours and external factors such as self-efficacy and social influences create *strong situations* and this in turn weakens the relationship between attitude and behaviours [22]. In short, attitudes tend to predict behaviour better in *weak situations* than in strong ones.

Second, students might hold a *non-responsive* attitude or affection that is acquired or learned from the existing values or social norms. For instance, ITN may be most commonly liked in the area simply for some reason other than its actual benefits. Such a situation may also happen if attitude questions in the survey were understood more as affection rather than objectively defined benefits and this was explained in observational learning theory (the modelling effects). It was also indicated that attitudes predict behaviour to the extent that attitude and behaviour are measured at the same level of specificity [22]. However, this complex relationship must be further explored.

## Limitation of the study

For this study, information on a parasitological survey which might have added value to the findings was not collected. Arguably, the choice of outcome measures for health intervention is critical in that health outcomes should be considered the index of success of interventions designed to change health behaviours [46]. However, it is believed that changes in behavioural measures, such as sustained use of ITN, are supposed to be a proxy indicator of health outcomes (i.e. reduced malaria infections). Thus, objective analysis of behavioural outcomes could be informative when evaluating behaviour change interventions.

Furthermore, limitation inherently to the unmatched design employed in the current study might have introduced bias that affects the result. However, an advanced statistical technique: the PSM analysis was applied as matching analysis to remove the possible confounding bias arising from the lack of matching so that the average effects of the intervention were easily estimated [31].

On top of this, different sources of biases for this study were also considered and dealt with. For instance, the possibility of information contamination among the control groups due to human mobility and interaction is often difficult to control in communication interventions. Furthermore, performance bias and social desirability bias (SDB) due to the self-reported behaviours might have also affected the data. Presumably, the SDB may be high among the intervention group since the population under study was already aware of the aim of the intervention and could respond to the interview in a socially desirable manner; thus hiding underlying reality. The measure of SDB score was controlled during the subsequent analysis to effectively estimate the averaged effects of the intervention. The data collectors were kept blind about the intervention and the target settings to control for possible performance bias.

## Conclusion

This study suggested the school-based SBCC approach advanced the malaria-related knowledge, attitude, self-efficacy, risk perceptions, and family supports, which have ultimately improved the sustained use of ITNs among school-going children. Schools' involvement through the integrated peer education and SBCC approaches would result in effective behaviour changes in schools regarding malaria preventions and beyond, which in turn helps to accelerate malaria elimination efforts. Furthermore, research should be conducted to broadly understand the mechanism by which the SBCC approach affects students’ behaviours given the influences of social, health services, and school systems.

## References

[1] WHO. Malaria vector control: Report of a WHO Study Group. Geneva, World Health Organization, 2006. (WHO Technical Report Series, 936). 16623084

[2] WHO (2020). World Malaria Report: Year of global progress and challenges.

[3] Autino B, Noris A, Russo R, Castelli F (2012). Epidemiology of malaria in endemic areas. Mediterr J Hematol Infect Dis.

[4] USAID and CDC: President malaria initiative, Ethiopia. Malaria operational plan 2019. Addis Ababa, Ethiopia; 2019.

[5] Walldorf JA, Cohee LM, Coalson JE, Bauleni A, Nkanaunena K, Kapito-Tembo A (2015). School-age children are a reservoir of malaria infection in Malawi. PLoS One.

[6] Pullan RL, Bukirwa H, Staedke SG, Snow RW, Brooker S (2010). *Plasmodium* infection and its risk factors in eastern Uganda. Malar J.

[7] Yeka A, Nankabirwa J, Mpimbaza A, Kigozi R (2015). Factors associated with malaria parasitemia, anemia and serological responses in a spectrum of epidemiological settings in Uganda. PLoS ONE.

[8] WHO (2019). Guidelines for malaria vector control.

[9] WHO (2016). Global technical strategy for malaria 2016–2030.

[10] RBM Partnership to end Malaria. The strategic framework for malaria social and behaviour change communication 2018–2030. Geneva, 2018.

[11] USAID and CDC. President malaria intitiative Ethiopia. Malaria Operational Plan FY 2020. Addis Ababa, Ethiopia; 2020.

[12] Taffese H, Hemming-Schoeder E, Koepfli C, Tesfaye G, Lee MC, Kazura J (2018). Malaria epidemiology and interventions in Ethiopia from 2001 to 2016. Infect Dis Poverty.

[13] USID/FHI 360. A Learning Package for Social and Behavior Change Communication (SBCC): Communication for Change (C‐Change) Project Version 3. Washington DC, USA; 2012.

[14] Brooker S, Kolaczinski JH, Gitonga CW, Noor AM, Snow RW (2009). The use of schools for malaria surveillance and programme evaluation in Africa. Malar J.

[15] Nonaka D, Kobayashi J, Jimba M, Vilaysouk B (2008). Malaria education from school to community in Oudomxay province. Lao PDR Parasit Int.

[16] Ayi I, Nonaka D, Adjovu JK, Hanafusa S, Jimba M, Bosompem KM (2010). School-based participatory health education for malaria control in Ghana : engaging children as health messengers. Malar J.

[17] Nyunt MH, Aye KM, Kyaw MP, Wai KT, Oo T, Than A (2015). Evaluation of the behaviour change communication and community mobilization activities in Myanmar artemisinin resistance containment zones. Malar J.

[18] Awantang G, Babalola S, Koenker H, Fox K, Toso M, Lewicky N (2018). Correlates of social behavior change communication on care-seeking behaviors for children with fever : an analysis of malaria household survey data from Liberia. Malar J.

[19] C-Change Final Report [Internet]. USAID, Washington DC; 2013. Available from: www.c-changeproject.org/sites/default/files/C-Change-Final-Report.March2013.pdf

[20] C-Change Project. Social and behavior change communication (SBCC). USAID, Washington DC;2012.

[21] Maddux JE, Rogers RW (1983). Protection motivation theory and self-efficacy: a revised theory of fear appeals and attitude change. J Exp Soc Psychol.

[22] Rogers EM, Everett M (1962). Diffusion of innovation.

[23] Kebede Y, Abebe L, Alemayehu G, Sudhakar M, Birhanu Z (2020). School-based social and behavior change communication (SBCC) advances community exposure to malaria messages, acceptance, and preventive practices in Ethiopia: a pre- posttest study. PLoS One.

[24] White H, Sabarwal S. Quasi-experimental design and methods: methodological briefs. Impact Evaluation 8, UNICEF. 2014;8. http://www.unicef-irc.org/KM/IE/

[25] Dattalo P (2008). Determining sample size balancing power, precision, and practicality.

[26] Ashton RA, Kefyalew T, Tesfaye G, Pullan RL, Yadeta D, Reithinger R (2011). School-based surveys of malaria in Oromia Regional State, Ethiopia: a rapid survey method for malaria in low transmission settings. Malar J.

[27] Central Statistical Agency (2016). Ethiopia Demographic and Health Survey 2016.

[28] Institute EPH (2016). Ethiopia national malaria indicator survey 2015.

[29] Ovadje L, Nriagu J (2016). Multi-dimensional knowledge of malaria among Nigerian caregivers : implications for insecticide-treated net use by children. Malar J.

[30] Patricia HM, Suzanne D, Baxter JAR (2015). Children's social desirability: effects of test assessment mode. Pers Individ Dif..

[31] Caliendo M, Kopeinig S. Some practical guidance for the implementation of propensity score matching. IZA Discussion paper 1588, 2005.

[32] Yao XI, Wang X, Speicher PJ, Hwang ES, Cheng P, Harpole DH (2017). Reporting and guidelines in propensity score analysis : a systematic review of cancer and cancer surgical studies. J Natl Cancer Inst..

[33] Boulay M, Lynch M, Koenker H (2014). Comparing two approaches for estimating the causal effect of behaviour-change communication messages promoting insecticide-treated bed nets : an analysis of the 2010 Zambia malaria indicator survey. Malar J.

[34] Cochran WG, Rubin DB, Sankhyā S, Indian T, Series A, Cochean BWG (2020). Controlling bias in observational studies: a review controlling bias in observational studies. Indian J Stat Ser A.

[35] Swain S, Pati S (2019). 'Health Promoting School’ model in prevention of vector-borne diseases in Odisha: a pilot intervention. J Trop Pediatr.

[36] Save the Children, National Malaria Control Programme, National Institute for Public Health Research, London school of Hygiene & Tropical Medicine. Malaria control in schools in Mali : Results from a cluster randomized control trial in Sikasso Region, Mali. Save the Children Resource Centre, 2012. https://resourcecentre.savethechildren.net/node/16064/pdf/malaria-control-mali.pdf.

[37] Sureshbabu J, Vasudevan S, Raj P (2017). A study of the effectiveness of school health education programs on selected mosquito borne diseases : school based cross-sectional study. Int J Res Med Sci.

[38] Rosenstock I (1974). Historical origins of the Health Belief Model. Health Education Monograph.

[39] Bandura A (1977). Self-efficacy: toward a unifying theory of behavioral change. Psychol Rev.

[40] Langford R, Bonell C, Jones H, Pouliou T, Murphy S, Waters E (2015). The World Health Organization’s Health Promoting Schools framework: a Cochrane systematic review and meta-analysis. BMC Public Health.

[41] Buchwald AG, Walldorf JA, Cohee LM, Coalson JE, Chimbiya N, Bauleni A (2016). Bed net use among school-age children after a universal bed net campaign in Malawi. Malar J.

[42] Birhanu Z, Abebe L, Sudhakar M, Dissanayake G, Yihdego Y (2015). Access to and use gaps of insecticide- treated nets among communities in Jimma Zone, southwestern Ethiopia : baseline results from malaria education interventions. BMC Public Health.

[43] Umwangange ML, Chironda G, Mukeshimana M (2018). Knowledge, attitude and practice towards malaria prevention among school children aged 5–14 years in sub-Saharan Africa - a review of Literature. Rwanda J Med Health Sci.

[44] Merakou K, Kourea-Kremastinou J (2006). Peer education in HIV prevention : an evaluation in schools. Eur J Public Health.

[45] Wallace DS, Paulson M, Lord CG, CFB. (2005). Which behaviors do attitudes predict ? Meta-analyzing the effects of social pressure and perceived difficulty. Rev Gen Psychol.

[46] Michie S, Abraham C (2004). Interventions to change health behaviours : evidence-based or evidence-inspired?. Psychol Health.

